# EvoDiffMol: evolutionary diffusion framework for 3D molecular design with optimized properties

**DOI:** 10.1186/s13321-026-01237-y

**Published:** 2026-07-15

**Authors:** Xiaobo Lin, Logan T. Kearney, Zhaoqian Su, Yunchao Liu, Amit K. Naskar, Debsindhu Bhowmik

**Affiliations:** 1 Carbon and Composites Group, Chemical Sciences Division, Oak Ridge National Laboratory, Oak Ridge, TN 37831 USA; 2 Research, Takeda Pharmaceutical Company Ltd., Cambridge, MA 02139 USA; 3 Broad Institute of MIT and Harvard, Cambridge, MA 02142 USA; 4 Computational Sciences and Engineering Division, Oak Ridge National Laboratory, Oak Ridge, TN 37831 USA

**Keywords:** Inverse molecular design, Diffusion models, Genetic algorithms, 3D molecular generation, Property optimization, Structural constraints

## Abstract

Designing molecules with specific target properties remains a fundamental challenge in computational chemistry. While existing approaches show promise, most rely on simplified representations like SMILES strings or 2D graphs that lack essential three-dimensional geometric information. We present EvoDiffMol, a computational framework that integrates evolutionary algorithms with three-dimensional diffusion models for property-driven molecular generation. The method operates through adaptive evolutionary optimization, where population-based selection guides the generation process toward desired property landscapes. EvoDiffMol supports both unconstrained molecular design and scaffold-constrained generation that preserves fixed substructures while optimizing complementary regions. Comprehensive evaluation demonstrates exceptional performance, achieving the highest drug-likeness score (0.94) among all compared state-of-the-art methods while maintaining excellent validity, uniqueness, and novelty. Beyond single property optimization, the framework demonstrates flexible multi-property optimization capabilities, simultaneously controlling multiple molecular descriptors including synthetic accessibility, lipophilicity, topological polar surface area, and clinically relevant ADMET properties such as cardiotoxicity (hERG) and intestinal permeability (Caco-2). This adaptability spans from simple descriptors to practical pharmaceutical endpoints without requiring complete model retraining. The framework achieves precise control over target property values, generating molecules with properties closely matching specified targets for both single and multiple descriptors. Scaffold-constrained experiments preserve fixed molecular cores while maintaining effective property optimization. The three-dimensional representation offers advantages in maintaining structural validity during iterative optimization, with potential for geometry-aware applications in materials science and drug discovery.

**Graphical abstract:**

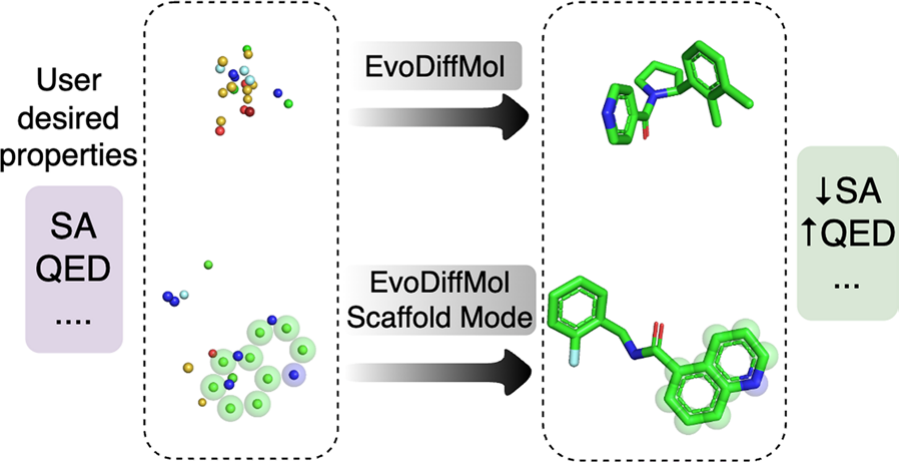

**Supplementary Information:**

The online version contains supplementary material available at 10.1186/s13321-026-01237-y.

## Scientific contribution

This work presents a novel framework that integrates adaptive evolutionary optimization with 3D equivariant diffusion models, enabling flexible multi-property and scaffold-constrained molecular design without retraining the generative model for new property objectives.

**Supplementary Information:**

The online version contains supplementary material available at 10.1186/s13321-026-01237-y.

## Introduction

Computational molecular design has emerged as a critical discipline bridging chemistry, biology, and materials science, driven by the need to discover compounds with precisely tailored characteristics [21, 25, 40, 49, 59]. The astronomical scale of chemical space, estimated to contain between $10^{23}$ and $10^{60}$ potentially druglike compounds while only approximately $10^8$ have been experimentally characterized [7, 52, 58], underscores the necessity for sophisticated computational strategies. Conventional discovery paradigms, which depend heavily on experimental screening and expert chemical intuition [23, 69], cannot adequately explore this vast molecular landscape.

The emergence of machine learning methodologies has transformed molecular generation through advanced generative modeling techniques [12, 22, 44, 59, 68]. Numerous contemporary approaches employ Simplified Molecular Input Line Entry System (SMILES) notation [66], treating molecular structures as textual sequences amenable to natural language processing techniques. Transformer-based architectures, including BERT variants [15, 19] and generative pre-trained models [2, 39], have demonstrated considerable success in generating syntactically correct molecular strings. Nevertheless, sequence-based representations impose fundamental constraints: they frequently generate chemically invalid structures [24, 52, 61, 62], omit crucial three-dimensional geometric information, and inadequately represent stereochemical features essential for biological activity and materials properties.

Diffusion models represent a paradigm shift in generative modeling, offering direct manipulation of three-dimensional molecular coordinates [27, 65, 70]. These approaches learn to progressively denoise molecular structures, generating geometrically consistent molecules while preserving essential spatial relationships and stereochemical information. The geometric awareness of diffusion processes offers potential advantages for representing molecular structure–property relationships, as steric interactions and chemical reactivity are influenced by three-dimensional atomic arrangements and interatomic distances. However, we note that the benefits of 3D representations depend on conformer quality and a single generated structure may not capture the full conformational ensemble relevant to thermodynamic properties.

While unconstrained molecular generation is important for exploring novel chemical space, generating molecules with fixed substructures or scaffolds while optimizing other molecular regions is also critical for practical applications [37, 43, 45]. Despite this importance, there has been limited research in scaffold-based molecule design, and even less work has focused on combining scaffold constraints with property optimization. This scaffold-constrained generation is essential in both drug discovery and materials science. In drug discovery, pharmaceutical companies need to maintain known active cores while modifying peripheral groups to improve properties like solubility, selectivity, or metabolic stability [10, 13, 64]. In materials science, preserving functional motifs while tuning other molecular features enables systematic property optimization [16, 31]. Traditional sequence-based methods struggle with scaffold constraints due to their limited ability to enforce structural preservation during generation [2, 45], highlighting the need for approaches that can handle both structural constraints and property optimization simultaneously.

Population-based optimization strategies, particularly evolutionary algorithms, have gained renewed attention in molecular design due to their competitive performance and inherent interpretability [8, 35, 42, 54, 72]. These approaches offer distinct advantages through their ability to define and control the chemical search space via customizable operators and rules. Modern evolutionary methods increasingly employ molecular-level mutations that enable smoother navigation of chemical space, facilitated by computational chemistry toolkits that streamline implementation and molecular validation. The integration of evolutionary principles with generative modeling offers compelling opportunities for property-driven molecular discovery while maintaining transparency in the optimization process. Recent work has demonstrated the effectiveness of adaptive training strategies for molecular language models [9] and reinforcement learning-based frameworks for controllable inverse molecular design [46], establishing a methodological progression toward combining adaptive optimization with generative molecular models.

We present EvoDiffMol, a comprehensive framework that synergistically combines adaptive evolutionary algorithms with three-dimensional diffusion models for targeted molecular design. Our approach exploits the geometric fidelity of diffusion-based generation while leveraging evolutionary optimization to guide molecular populations toward desired property landscapes. The system accommodates both unconstrained molecular generation and scaffold-preserving design scenarios. The key contributions of this work include:

1. A framework that integrates 3D diffusion models with evolutionary algorithms to generate and optimize molecular structures for desired properties, achieving the highest drug-likeness score (QED = 0.94) among state-of-the-art methods while demonstrating superior quality metrics across validity, uniqueness, and novelty.
2. A flexible optimization architecture that supports both single and multi-property objectives (including ADMET properties) based on user specifications, without requiring complete model retraining when targeting new molecular properties.
3. A dual-mode generation capability that accommodates both unconstrained molecular design and constrained generation with a desired fixed substructure, enabling property optimization while preserving essential chemical scaffolds.

**Fig. 1:**
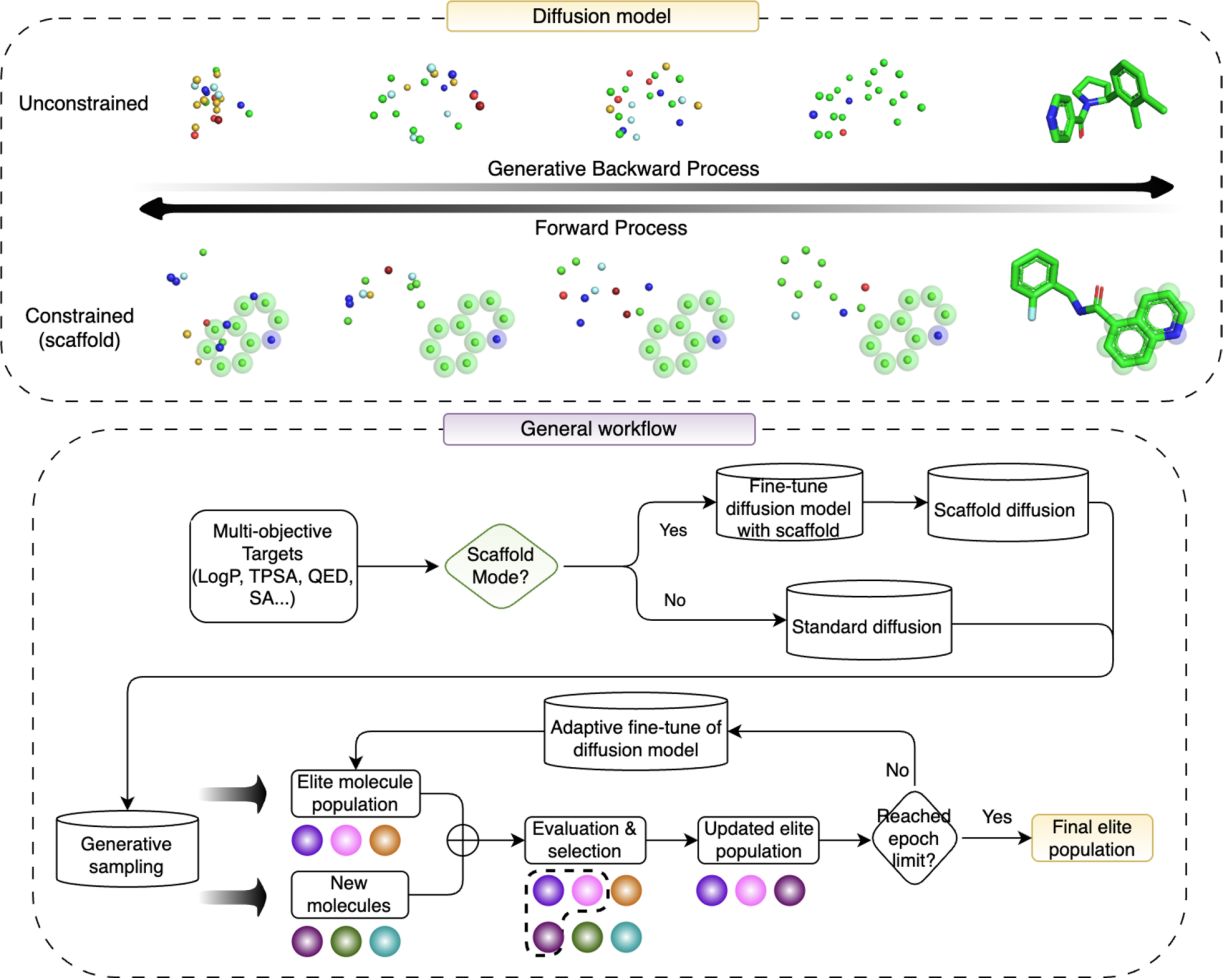
Diffusion–evolution workflow overview for 3D molecular design. Top: The diffusion model corrupts and then denoises molecular structures, enabling generative backward sampling for either unconstrained design (top row) or scaffold-constrained design (bottom row; green halos indicate fixed scaffold atoms). Bottom: Users specify one or more property targets (e.g., LogP, TPSA, QED, SA, ADMET properties). Depending on "Scaffold Mode," the model runs standard diffusion or is fine-tuned with the given scaffold, performs generative sampling, and scores candidates

## Results and discussion

This section presents a comprehensive analysis of EvoDiffMol’s performance across diverse molecular design challenges. We begin by benchmarking the framework against state-of-the-art molecular generation methods, achieving superior drug-likeness optimization (QED = 0.94) while maintaining excellent validity, uniqueness, and novelty. We then demonstrate the framework’s multi-property optimization capabilities in unconstrained molecular generation, showing simultaneous control over multiple molecular descriptors. Finally, we extend the evaluation to scaffold-constrained design, where property optimization is performed while preserving fixed molecular substructures. The results demonstrate the synergistic benefits of integrating evolutionary optimization with three-dimensional diffusion models for creating high-quality molecular libraries with targeted characteristics.

### Framework overview

Figure 1 illustrates the operational architecture of EvoDiffMol, which employs an iterative, population-based optimization strategy that synergizes a three-dimensional diffusion model with an adaptive evolutionary algorithm. The framework begins with a pre-trained diffusion model and a user-defined set of property objectives. Its core is a generate–evaluate–adapt loop designed to progressively steer the molecular generation process toward regions of chemical space that satisfy the desired criteria.

Importantly, EvoDiffMol’s evolutionary optimization differs from classical genetic algorithms in that it does not employ explicit crossover or mutation operators on molecular structures. Instead, structural variation is introduced through stochastic sampling from the diffusion model, and evolution proceeds via fitness-based selection of elite candidates combined with adaptive fine-tuning of the generative model on selected populations. The diffusion model itself serves as the variational operator, generating diverse molecular candidates at each generation.

The evolutionary cycle begins with an initial population of 3D molecules sampled from the diffusion model. In the evaluation phase, each molecule is assessed using a scoring function that calculates the desired chemical properties (e.g., LogP, TPSA, SA). A composite fitness score is then computed for each candidate, typically using a harmonic mean to balance multiple, often competing, objectives. Based on these scores, an elite subset of the highest-performing molecules is selected. In the adaptation phase, the diffusion model’s weights are updated by fine-tuning exclusively on this elite population. This adaptive training progressively biases the model to generate molecules with characteristics similar to the top performers, creating a feedback loop where high-performing molecules become the training data for the next generation. The newly adapted model is then used to generate the next population of molecules, and the cycle repeats.

The framework supports two distinct operational modes: unconstrained and scaffold-constrained generation. In the unconstrained mode, the process begins directly with the pre-trained diffusion model. For scaffold-constrained tasks, an additional pre-processing step is introduced. Before the main evolutionary loop, the base diffusion model is initially fine-tuned on a filtered dataset containing molecules with the specified scaffold. This scaffold-aware fine-tuning enables the model to learn how to generate molecules when provided with scaffold context, effectively creating a conditional generative model that can incorporate fixed substructures as input context. The subsequent generate–evaluate–adapt cycles then operate using this scaffold-aware model, ensuring that all generated molecules preserve the core scaffold while the evolutionary algorithm optimizes the appended functional groups for the target properties.

### Molecular generation

High performance in validity, uniqueness, and novelty metrics is essential for molecular generation, ensuring that models successfully learn molecular structure patterns while avoiding overfitting to training data [24, 27, 52, 61]. The diffusion model is pre-trained on the MOSES dataset [56] to learn the joint distribution of 3D atomic coordinates and discrete atom types within molecular graphs, serving as the foundation for property-directed molecular design. The framework supports flexible property optimization using various molecular descriptors such as synthetic accessibility (SA), drug-likeness (QED), lipophilicity (LogP), topological polar surface area (TPSA), and ADMET properties (cardiotoxicity, intestinal permeability), which can be targeted individually or in combination based on specific design objectives, and can be readily extended to incorporate additional molecular properties.

#### Comparison with state-of-the-art methods

We compare EvoDiffMol’s QED optimization performance with other state-of-the-art methods that target drug-likeness maximization, where higher QED values (closer to 1) indicate better drug-like properties. Table 1 presents this comprehensive comparison against established generative modeling approaches, with comparison methods’ results obtained from the Taiga study [52], which evaluated these methods on the MOSES dataset under standardized conditions, ensuring a fair comparison. The comparison encompasses multiple methodological paradigms: (1) GCPN [73]: employs reinforcement learning through proximal policy optimization combined with adversarial training on graph representations; (2) JTVAE [36]: utilizes junction tree decomposition within a variational autoencoder framework for graph generation; (3) MolGPT [2]: applies transformer architectures for sequential SMILES generation; (4) GraphDF [48]: implements discrete normalizing flows for molecular generation; (5) LSTM [52, 57]: uses policy gradient reinforcement learning for sequence-based generation; and (6) Taiga [52]: molecule generation using transformers and policy gradient reinforcement learning. All comparison methods were evaluated for QED optimization using their released code and respective optimization strategies, as reported in the Taiga study [52]. These include reinforcement learning with QED-based rewards (GCPN, LSTM, Taiga), Bayesian optimization in latent space (JTVAE), conditional generation (MolGPT), and reward-guided normalizing flows (GraphDF). In contrast, EvoDiffMol uses evolutionary selection on diffusion-generated populations, where the generative model is adaptively fine-tuned on elite molecules rather than optimized through reward signals. EvoDiffMol leverages three-dimensional atomic coordinates and molecular graphs, converting to SMILES representations exclusively for performance metric evaluation, molecular property computation, and fitness scoring.

**Table 1:** Performance metrics for QED-optimized molecular generation

Method	QED	Validity (%)	Uniqueness (%)	Novelty (%)
*SMILES-based methods*				
GCPN$^{\dagger }$	0.64 ± 0.15	99	99	99
JTVAE$^{\dagger }$	0.70 ± 0.12	100	100	100
MolGPT$^{\dagger }$	0.75 ± 0.11	62	98	99
GraphDF$^{\dagger }$	0.42 ± 0.13	100	99	99
LSTM$^{\dagger }$	0.80 ± 0.07	95	90	82
Taiga$^{\dagger }$	0.83 ± 0.07	97	99	95
*3D diffusion baseline*				
MDM/EvoDiffMol base	0.79 ± 0.10	99	97	96
*After evolutionary optimization*				
EvoDiffMol	0.94 ± 0.01	100	100	95

$^{\dagger }$Results from Taiga study [52], evaluated on MOSES dataset under comparable conditions. See Supporting Information for detailed experimental configurations

**Fig. 2:**
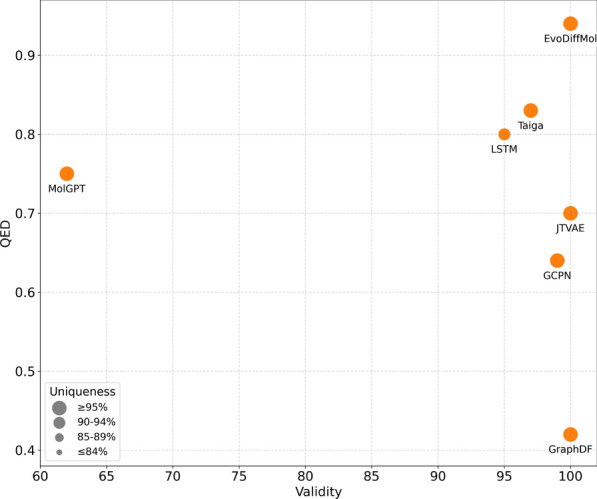
Comparison of molecular generation methods on MOSES dataset. Scatter plot shows QED versus validity for different methods, with dot size representing uniqueness (see legend)

Table 1 presents a comprehensive comparison of EvoDiffMol against state-of-the-art molecular generation methods, revealing fundamental differences in how various approaches balance drug-likeness optimization with structural quality. Among SMILES-based methods, a clear trade-off pattern emerges: approaches prioritizing QED optimization often sacrifice validity, while those ensuring validity achieve lower QED scores. Taiga represents the best SMILES-based performance with QED of 0.83 ± 0.07, followed by LSTM (0.80 ± 0.07), though LSTM shows reduced uniqueness (90%) and novelty (82%), suggesting potential mode collapse or training data memorization. MolGPT achieves intermediate QED (0.75 ± 0.11) but suffers from critically low validity (62%), rendering nearly 40% of generated molecules chemically invalid and unusable. Conversely, methods ensuring high validity (JTVAE, GCPN, GraphDF) produce substantially lower QED values (0.42−0.70), demonstrating the difficulty of simultaneously optimizing drug-likeness while maintaining chemical validity in sequence-based representations.

The baseline 3D diffusion model (MDM/EvoDiffMol base) without evolutionary optimization achieves QED of 0.79 ± 0.10 with 99% validity, 97% uniqueness, and 96% novelty, demonstrating that 3D geometric representations inherently support robust generation of chemically sound, diverse, and novel structures. This baseline already matches or exceeds most SMILES methods in validity and uniqueness while maintaining competitive QED. Evolutionary optimization dramatically enhances this foundation, pushing QED from 0.79 to 0.94±0.01 with minimal variance, indicating tight convergence around optimal drug-like properties. The optimized population achieves 100% validity and uniqueness throughout all generations while preserving high novelty (95%), confirming the framework generates genuinely new molecules rather than memorizing training data. This combination of highest QED, perfect validity, and maintained novelty represents a breakthrough in balancing multiple competing objectives that typically require trade-offs in other approaches.

Figure 2 visualizes this performance landscape across QED and validity dimensions, with dot size encoding uniqueness. EvoDiffMol occupies the upper-right corner, representing the only method achieving both highest drug-likeness and perfect structural validity.

Table 1 implicitly compares SMILES-based generators with optimization (Taiga, LSTM+RL) against our 3D diffusion generator with evolutionary optimization. The Generation 0 baseline (Table 2) further isolates the evolutionary contribution: the diffusion model alone achieves QED = 0.79, while evolutionary optimization raises this to 0.94, a +19% improvement. This indicates that the evolutionary optimization is the primary driver of property improvement, while the 3D diffusion backbone contributes to structural quality—maintaining 100% validity throughout optimization compared to 62–99% for SMILES-based methods. We attribute this validity advantage to the geometric constraints inherent in 3D coordinate-based generation, which naturally enforce chemically reasonable bond lengths and angles. A fully controlled ablation using identical evolutionary frameworks with different generator backbones represents a valuable direction for future work.

#### Evolutionary trajectory and diversity maintenance

A critical concern in iterative fine-tuning approaches is whether repeated model adaptation on elite populations causes mode collapse, where the model progressively narrows its output distribution and loses structural diversity. To investigate this, we tracked fitness and diversity metrics throughout the evolutionary optimization process. Table 2 presents QED progression alongside two complementary diversity measures across generations 0, 1, 3, 5, and 10: Tanimoto similarity (molecular fingerprint diversity) and scaffold diversity (structural framework variety).

**Table 2:** Ablation study: QED optimization trajectory and diversity maintenance across evolutionary generations

Generation	QED (Fitness)	Tanimoto similarity	Scaffold diversity (%)
0 (baseline)	0.79 ± 0.10	0.14	84
1	0.89 ± 0.03	0.16	71
3	0.92 ± 0.01	0.18	70
5	0.93 ± 0.01	0.17	73
10 (final)	0.94 ± 0.01	0.17	75

Tanimoto similarity: lower values indicate higher diversity. Scaffold diversity: percentage of unique Bemis-Murcko scaffolds. Multi-seed reproducibility (4 independent runs): QED = 0.936 ± 0.001 (between-run std), Tanimoto = 0.173 ± 0.002, scaffold diversity = 73.1% ± 1.2% (see Supporting Information Table S6)

**Fig. 3:**
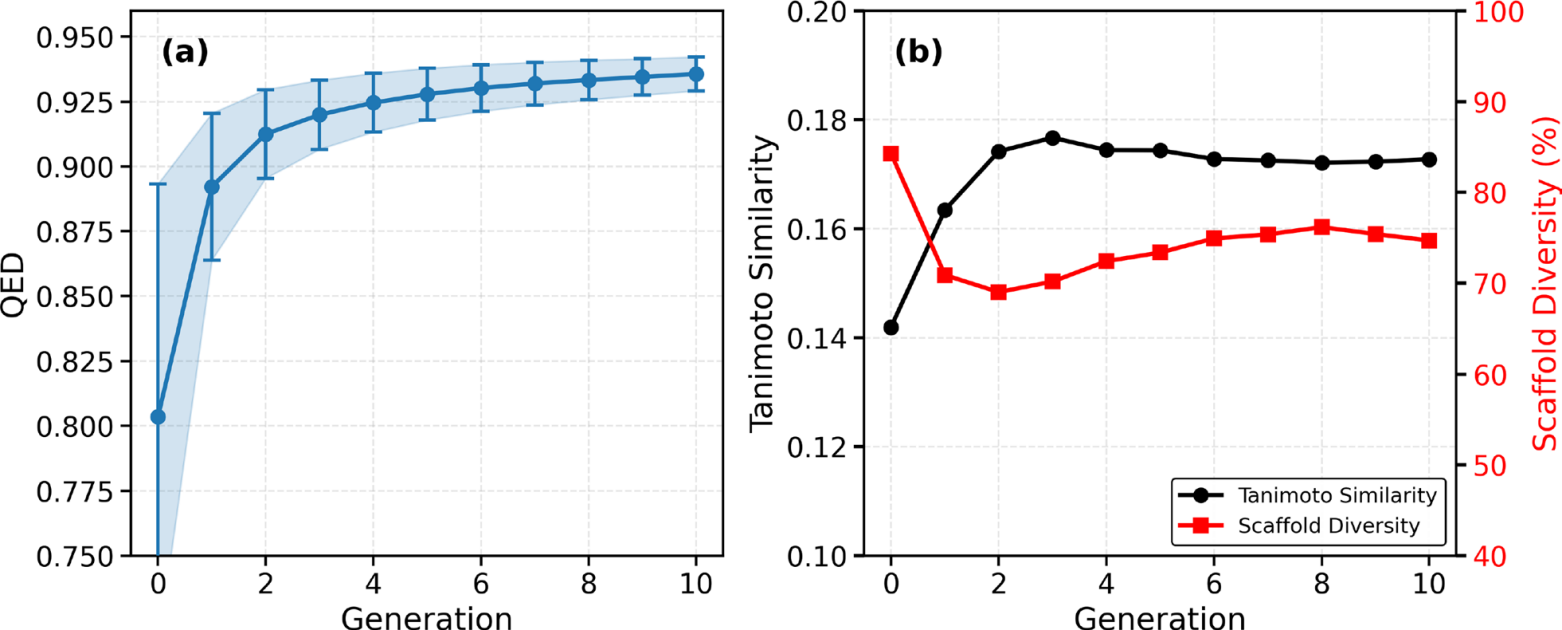
Evolutionary optimization trajectory for QED maximization. **a** QED progression from generation 0 (baseline) to generation 10, showing systematic improvement from 0.79 ± 0.10 to 0.94 ± 0.01. Error bars and shaded region represent standard deviations. **b** Molecular diversity metrics across generations: Tanimoto similarity (left axis, black) remains stable around 0.17, and scaffold diversity (right axis, red) remains high (70–84%)

Table 2 and Fig.3 reveal systematic QED improvement from 0.79 ± 0.10 (generation 0) to 0.94 ± 0.01 (generation 10), representing a 19.0% increase in drug-likeness. The optimization trajectory exhibits rapid initial convergence: QED jumps from 0.79 to 0.89 in generation 1, then reaches 0.92 by generation 3, with subsequent generations showing diminishing returns (0.93 at generation 5, 0.94 at generation 10). The standard deviation decreases from 0.09 to 0.01, indicating tight convergence around optimal drug-like properties. Critically, this fitness improvement occurs without sacrificing structural diversity: Tanimoto similarity remains stable around 0.17 (range: 0.14−0.18), and scaffold diversity remains high at 70–84% throughout all generations, with approximately 1,800 unique Bemis-Murcko scaffolds among 2,500 molecules. The modest decrease from 84% (generation 0) to 75% (generation 10) reflects convergence toward preferred drug-like structural frameworks rather than mode collapse, as confirmed by the stable Tanimoto similarity.

The maintained high scaffold diversity confirms that evolutionary optimization explores diverse structural frameworks to achieve high QED, rather than exploiting a single privileged scaffold. This structural variety is crucial for practical drug discovery, as diverse scaffolds provide multiple starting points for lead optimization. Multi-seed experiments (4 independent runs) confirm that scaffold diversity (73.1% ± 1.2%) and Tanimoto similarity (0.173 ± 0.002) are reproducible across runs (Supporting Information Table S6).

#### Multi-property optimization

**Fig. 4:**
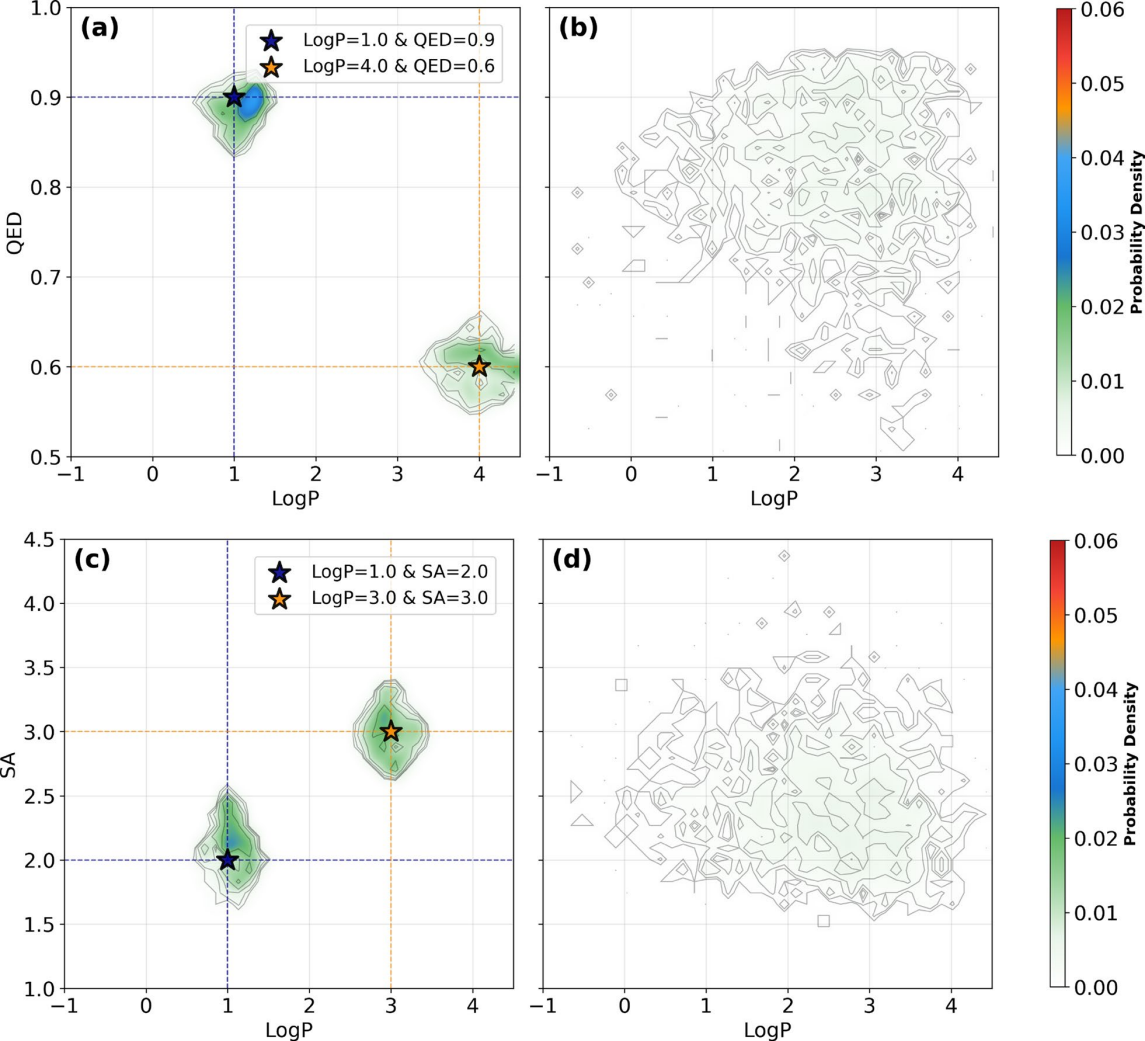
Pairwise property optimization for molecular generation. Contour plots show molecular distributions in two-dimensional property space with EvoDiffMol targeting specific coordinates (stars). **a** QED vs LogP optimization by EvoDiffMol, **b** MOSES dataset distribution for QED vs LogP, **c** SA vs LogP optimization by EvoDiffMol, **d** MOSES dataset distribution for SA vs LogP

**Fig. 5:**
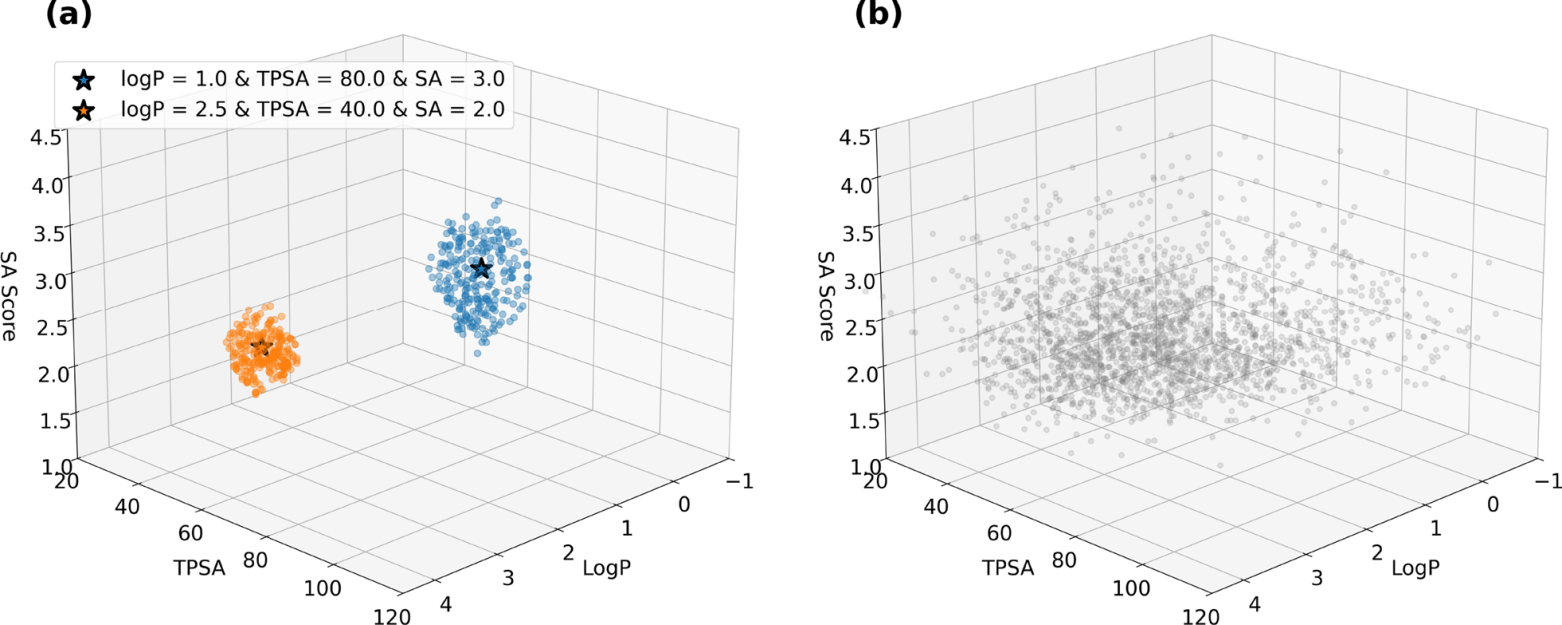
Triple property optimization for molecular generation. Three-dimensional scatter plots show molecular distributions in LogP, TPSA, and SA property space with EvoDiffMol targeting specific coordinates (stars). **a** EvoDiffMol optimization results, **b** MOSES dataset distribution

Beyond single-property optimization, EvoDiffMol enables precise multi-property control, addressing the practical reality that drug discovery requires simultaneous optimization of multiple, often competing molecular characteristics. Single-property optimization results (Supporting Information Figure S1) establish baseline performance: the framework successfully optimizes LogP, TPSA, SA, and QED individually, producing narrow distributions centered at specified targets across diverse property ranges, including challenging boundary targets with sparse prior density in the training distribution.

Figures 4 and 5 demonstrate bivariate and trivariate optimization capabilities. The bivariate analysis reveals tight control over property pairs: for LogP-QED optimization, generated molecules cluster around target coordinates (LogP, QED) $\approx$ (1.0, 0.9) and (4.0, 0.6), with target locations (dashed lines) and sample means (stars) aligning closely. In contrast, the MOSES test set exhibits diffuse, unconcentrated distributions across the property space. This pattern extends to LogP-SA optimization, demonstrating reliable multi-objective steering across qualitatively different property pairs: LogP-QED combines physicochemical and drug-likeness characteristics, while LogP-SA balances lipophilicity with synthetic accessibility. The ability to achieve tight clustering at diverse target locations (e.g., high QED with low LogP versus moderate QED with high LogP) indicates the framework navigates property space flexibly rather than exploiting fixed correlations in the training data.

Triple-property optimization (Fig. 5) extends control to three dimensions simultaneously. Generated molecules form compact clusters around target triplets (LogP, TPSA, SA) $\approx$ (1.0, 80, 3) and (2.5, 40, 2), marked by black stars, while the MOSES reference set exhibits widespread, unconcentrated distribution throughout the 3D property space. The successful positioning of molecular populations at prescribed coordinates in three-dimensional space demonstrates that evolutionary optimization effectively balances multiple competing objectives without sacrificing control over any individual property. This multi-dimensional control is essential for practical molecular design, where target profiles typically specify multiple property constraints simultaneously (e.g., drug-like molecules with specific lipophilicity, polar surface area, and synthetic accessibility).

#### ADMET property optimization

Beyond simple molecular descriptors, the framework accommodates clinically relevant ADMET (Absorption, Distribution, Metabolism, Excretion, Toxicity) properties as optimization targets, addressing practical drug discovery requirements. ADMET properties directly correlate with clinical success and represent concrete pharmaceutical endpoints that determine whether a molecule can become a viable drug candidate [20].

To demonstrate practical applicability, we simultaneously optimize four properties combining basic drug-likeness metrics with critical ADMET endpoints: QED (drug-likeness), SA (synthetic accessibility), hERG (cardiotoxicity) [14], and Caco-2 permeability (intestinal absorption) [29]. The optimization targets molecules with higher drug-likeness, easier synthesis (lower SA score), lower cardiotoxicity risk, and higher intestinal permeability. This combination represents a realistic drug discovery scenario where multiple competing objectives must be balanced: synthesizable, drug-like molecules with favorable safety and absorption profiles. We compare the optimized population against the MOSES dataset, which represents typical drug-like molecules from medicinal chemistry literature.

**Fig. 6:**
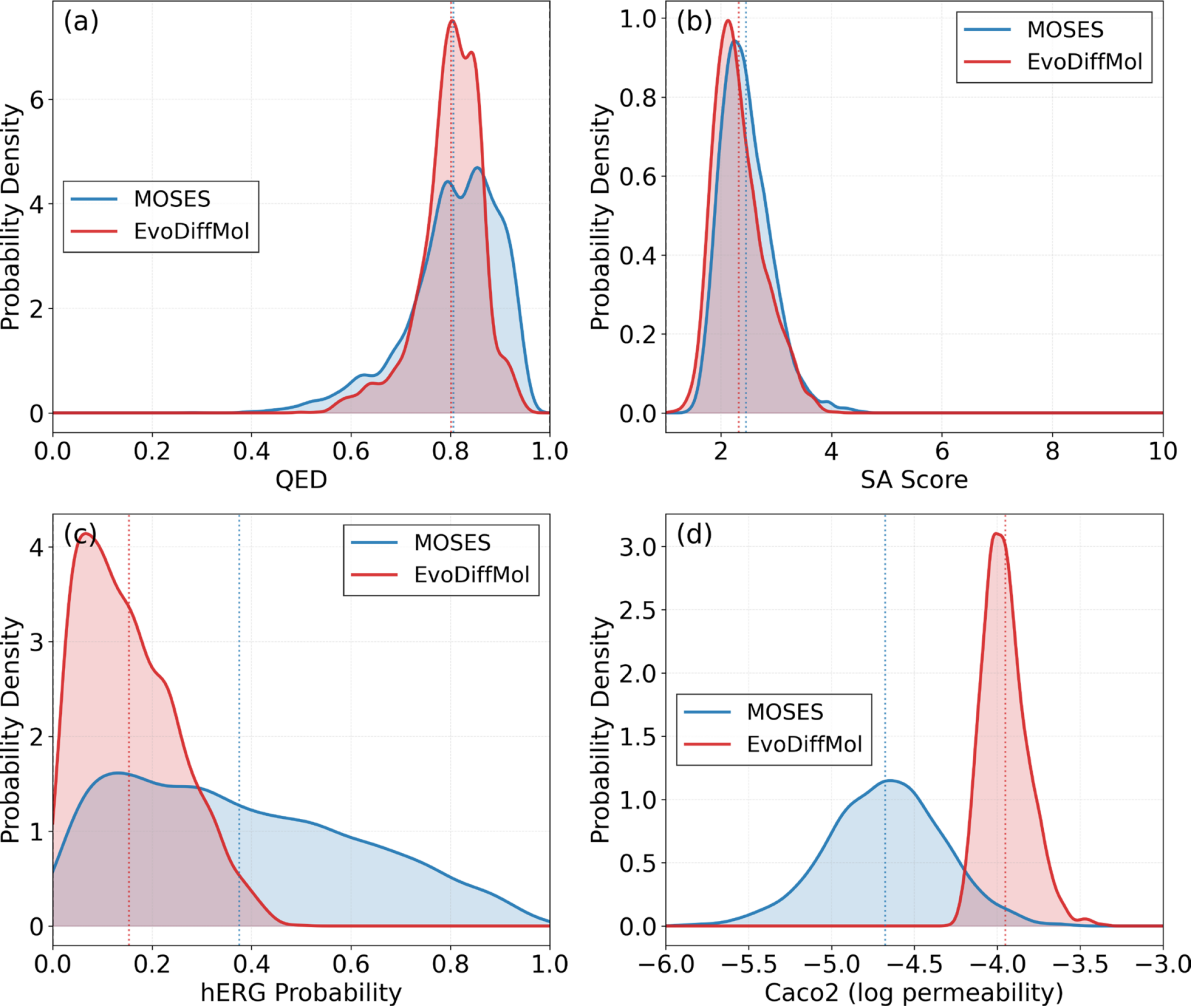
ADMET property optimization results comparing MOSES dataset (blue) with EvoDiffMol optimized population (red). Probability density distributions for: **a** QED (drug-likeness), **b** SA (synthetic accessibility), **c** hERG toxicity, **d** Caco-2 permeability. Black dashed lines indicate optimization targets. Dotted lines show mean values for each distribution. EvoDiffMol maintains favorable drug-like properties while dramatically improving ADMET profiles, particularly cardiotoxicity and permeability

**Table 3:** ADMET multi-property optimization: MOSES dataset versus EvoDiffMol

Property	MOSES	EvoDiffMol	Improvement
QED ($\uparrow$)	0.81 ± 0.09	0.80 ± 0.06	− 0.6%
SA ($\downarrow$)	2.45 ± 0.45	2.32 ± 0.45	+ 5.3%
hERG toxicity ($\downarrow$)	0.38 ± 0.24	0.15 ± 0.10	+ 59.1%
Caco-2 permeability ($\uparrow$)	$-4.68\pm 0.35$	$-3.95\pm 0.13$	+ 15.5%

hERG toxicity is a probability score ranging from 0 to 1, where lower values indicate reduced cardiotoxicity risk. Caco-2 permeability in log$_{10}$(P$_{\textrm{app}}$, cm/s). Improvement percentages indicate change relative to MOSES baseline

Figure 6 and Table 3 illustrate EvoDiffMol’s successful multi-property optimization compared to the MOSES dataset baseline. While MOSES molecules already exhibit favorable drug-likeness (QED: 0.81 ± 0.09) and synthetic accessibility (SA: 2.45 ± 0.45), they show suboptimal ADMET profiles. EvoDiffMol maintains these drug-like properties (QED: 0.80 ± 0.06, SA: 2.32 ± 0.45) while dramatically improving critical ADMET endpoints: cardiotoxicity risk (hERG toxicity) decreases by 59% from 0.38±0.24 to 0.15±0.10, and intestinal permeability (Caco-2, log$_{10}$(P$_{\textrm{app}}$, cm/s)) increases by 15.5% from $-4.68\pm 0.35$ to $-3.95\pm 0.13$. The maintained QED and SA values demonstrate that EvoDiffMol does not sacrifice drug-likeness or synthetic feasibility while optimizing ADMET properties. The dramatic reduction in hERG liability is particularly significant, as cardiotoxicity represents a major cause of drug attrition in clinical development. This multi-property optimization showcases the framework’s ability to navigate complex pharmaceutical design spaces with multiple competing objectives, achieving substantial improvements in safety and absorption profiles while preserving favorable drug-like characteristics.

Unlike simple molecular descriptors, ADMET properties represent measurable pharmacokinetic endpoints with direct clinical implications. While we demonstrate optimization using hERG (cardiotoxicity) and Caco-2 (intestinal permeability) as representative examples, the framework supports a comprehensive range of ADMET endpoints through machine learning predictions, enabling users to target specific pharmacokinetic properties relevant to their drug discovery objectives. The ability to simultaneously optimize drug-likeness, synthetic accessibility, and critical ADMET endpoints demonstrates the framework’s applicability to realistic drug development scenarios requiring balanced optimization of multiple pharmaceutical endpoints.

### Scaffold-constrained molecular design

Scaffold-constrained generation represents a critical capability in practical drug discovery, where specific molecular scaffolds must be preserved while optimizing complementary properties [43, 45, 47]. This section demonstrates EvoDiffMol’s scaffold-constrained optimization using quinoline as the primary example, with additional diverse scaffolds (pyridine, thiophene, piperazine) and ADMET property optimization presented in the Supporting Information.

**Fig. 7:**
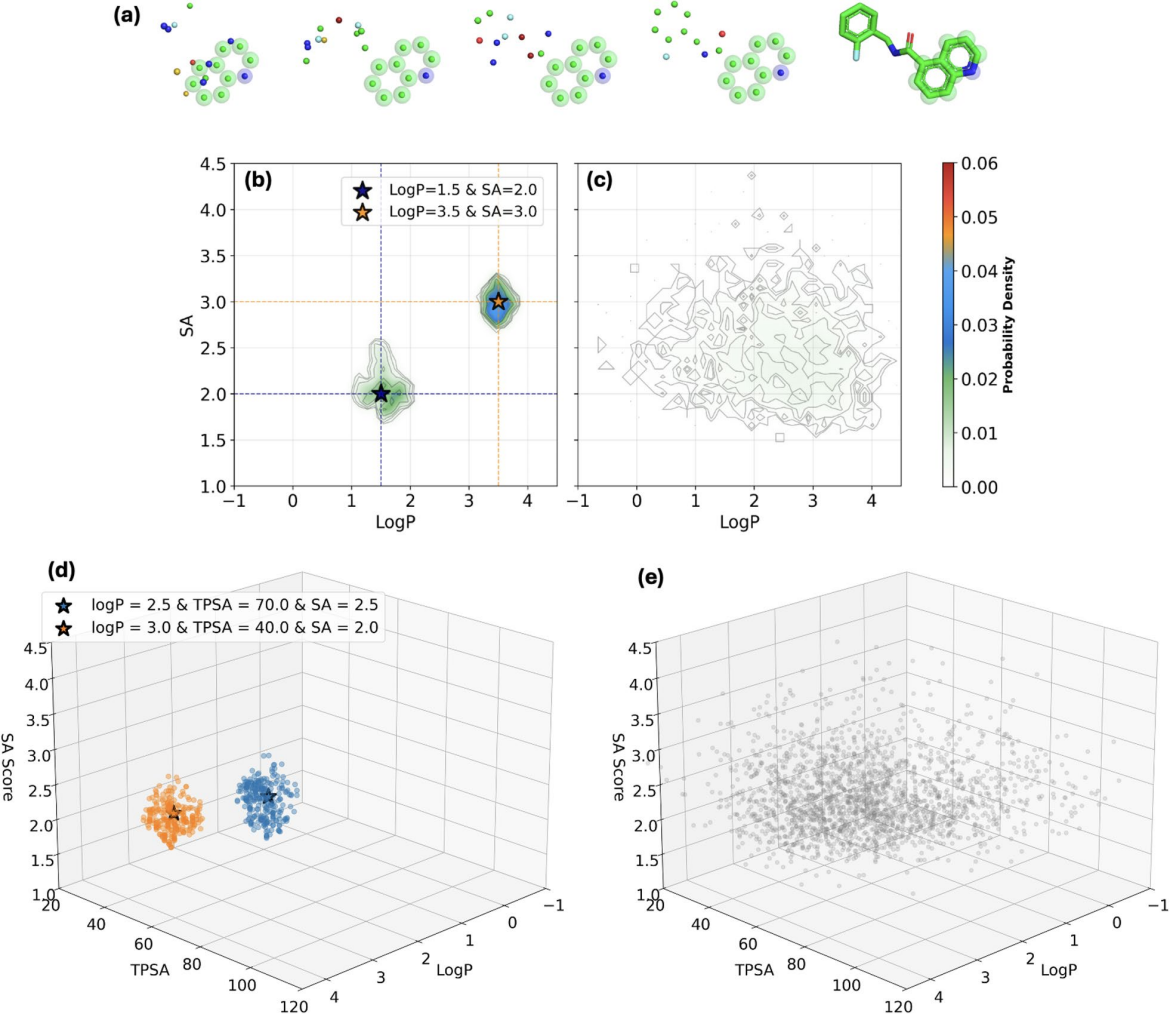
Multi-property optimization for scaffold-constrained molecular generation with quinoline scaffold. **a** Scaffold-constrained diffusion process: quinoline core (highlighted in green) remains fixed while surrounding atoms are progressively denoised from random noise to chemically valid structures. **b** LogP vs SA optimization by EvoDiffMol with two target combinations (stars). **c** MOSES dataset distribution for LogP vs SA. **d** Triple property (LogP, TPSA, SA) optimization by EvoDiffMol with two target triplets (stars). **e** MOSES dataset distribution in 3D property space

Quinoline represents a pharmaceutically privileged scaffold with established therapeutic relevance across multiple drug discovery programs [67]. This heterocyclic structure exhibits significant medicinal chemistry potential with a broad spectrum of biological activities [41, 51]. Our approach implements scaffold-conditioned diffusion where scaffold atoms remain fixed during the denoising process, while newly generated atoms are constrained to positions compatible with the existing scaffold structure (Fig. 7a). To enhance generation quality, the pre-trained diffusion model undergoes fine-tuning on filtered training data containing molecules with the target scaffold, improving the model’s ability to generate chemically coherent scaffold extensions. This fine-tuning strategy is applied consistently across all scaffold examples, including the ADMET optimization cases in the Supporting Information. Detailed methodology for scaffold-based diffusion is provided in the Methods section.

#### Scaffold-constrained property optimization

Scaffold-constrained generation requires simultaneous satisfaction of structural preservation and property optimization objectives, presenting greater challenges than unconstrained design. The framework’s 3D representation provides a key advantage: flexible molecular regions can spatially interact with the fixed scaffold’s geometry during generation, enabling geometric awareness that SMILES-based approaches cannot capture [27, 70].

Maintaining scaffold structural integrity throughout optimization is critical. Since bond perception is determined from 3D coordinates and atomic distances [55], newly generated atoms near the scaffold can potentially alter perceived connectivity. The framework incorporates filtering to remove molecules where scaffold connectivity has been disrupted, ensuring all optimized candidates retain the intended architecture.

Single-property optimization under quinoline scaffold constraints (Supporting Information Figure S2) demonstrates precise control over LogP, TPSA, SA, and QED, generating narrow distributions centered at user-specified targets. Multi-property optimization (Fig. 7) extends this control to simultaneous objectives. Bivariate optimization (panels b-c) produces compact density clusters around target pairs with clean separation between objectives, while triple-property analysis (panels d-e) shows well-separated, compact clouds around target triplets. Comparing these scaffold-constrained results to unconstrained optimization reveals comparable property control precision, indicating that the framework effectively navigates the reduced chemical space to discover multiple solutions satisfying both structural and property constraints without significant performance degradation.

Scaffold constraints fundamentally reduce available chemical space compared to unconstrained generation [2], as property optimization must occur exclusively through modifications to non-scaffold regions. Despite this challenge, EvoDiffMol achieves effective property control while maintaining scaffold integrity. The Supporting Information extends this demonstration to additional pharmaceutically relevant scaffolds (pyridine, thiophene, piperazine) optimizing drug-likeness (QED), synthetic accessibility (SA), and cardiotoxicity (hERG), confirming consistent performance across diverse structural templates and clinically relevant safety objectives. This extensibility demonstrates the framework’s practical utility for lead optimization workflows where maintaining known active scaffolds while improving drug-like properties and safety profiles is essential.

## Conclusion

EvoDiffMol presents a promising approach to computational molecular design through the integration of evolutionary algorithms with three-dimensional diffusion models. Our framework addresses limitations of sequence-based approaches by incorporating explicit geometric information for molecular property prediction and optimization. Comprehensive evaluation demonstrates competitive performance across diverse molecular design challenges, showing EvoDiffMol as an effective tool for targeted molecular discovery.

The framework achieves excellent performance across structural validity, uniqueness, novelty, and multi-property optimization, and can handle both unconstrained and scaffold-constrained generation scenarios. The evolutionary optimization component is the primary driver of property improvement, while the 3D diffusion backbone contributes to structural validity and generation quality during iterative fine-tuning.

Several critical innovations distinguish EvoDiffMol from existing methodologies: (1) integration of diffusion processes with evolutionary algorithms for three-dimensional molecular design, (2) flexible multi-property optimization supporting single, dual, and triple property targets without requiring complete model retraining, (3) scaffold-constrained generation with desired fixed substructures while maintaining property optimization capabilities, (4) exceptional performance in drug-likeness optimization, achieving the highest QED score (0.94) among all compared state-of-the-art methods while maintaining excellent validity, uniqueness, and novelty metrics, and (5) demonstration of practical pharmaceutical applications through simultaneous ADMET property optimization, successfully targeting clinically relevant endpoints such as cardiotoxicity and intestinal permeability alongside drug-likeness and synthetic accessibility.

Beyond current applications, the three-dimensional geometric capabilities uniquely position EvoDiffMol for advanced structure-based molecular design challenges. The explicit 3D coordinate representation enables future applications such as linker design for drug development [3, 30, 32], molecular docking studies [1, 11], and for traditional polymers, the framework could potentially be extended to design polymers with desired chain packing, crystallinity, and morphological features based on 3D structural principles [34, 37, 38, 53, 71, 74, 75].

The modular framework architecture enables easy incorporation of diverse molecular properties, as demonstrated through successful ADMET optimization. The framework’s ability to accommodate properties ranging from simple descriptors (LogP, TPSA) to complex pharmacokinetic endpoints (e.g., hERG cardiotoxicity, Caco-2 permeability) illustrates its adaptability to user-specific requirements. This flexibility supports diverse research applications where custom property combinations can be targeted without requiring fundamental changes to the underlying diffusion-evolution architecture.

While EvoDiffMol represents substantial progress in three-dimensional molecular design, several limitations warrant consideration. Computational requirements exceed those of simpler sequence-based approaches due to the complexity of three-dimensional geometric modeling and population-based optimization. Additionally, performance depends on the quality of underlying diffusion models and property prediction functions. The property evaluations in this study (QED, LogP, TPSA, SA, ADMET predictions) are computed from 2D molecular representations and do not directly assess 3D-dependent molecular behavior. The advantages of 3D representation in our framework manifest primarily in maintaining structural validity during iterative generation rather than in property optimization performance. Comprehensive 3D-sensitive evaluations—including molecular docking, conformer energy landscapes, and chirality recognition—represent important directions for future work.

For deterministic molecular descriptors (LogP, TPSA, SA, QED), property values are computed from mathematical formulas without learned parameters; using the same calculator for optimization and evaluation confirms the optimization achieves its defined objective. For ML-based ADMET predictions, a genuine circularity risk exists: evolutionary optimization can amplify predictor-specific biases through iterative selection. Evaluation across non-targeted ADMET endpoints (CYP2D6, DILI, BBB, HIA) shows consistent improvement (Supporting Information Table S4), suggesting the optimization produces generally favorable drug-like molecules rather than exploiting endpoint-specific artifacts. However, all predictions come from the same ADMET-AI framework, and different predictors may yield different results. ADMET optimization results should be interpreted as predictor-specific trends, and experimental validation remains essential for practical drug discovery applications.

All evaluations in this study use the MOSES benchmark, which represents a constrained drug-like chemical space (MW 250–350 Da, $\le$7 rotatable bonds, XlogP < 3.5). While this enables standardized comparison with prior methods, the framework’s generalization to larger, more diverse chemical libraries (e.g., ZINC, ChEMBL, GDB-17) remains to be validated. Evaluating EvoDiffMol across broader chemical domains is an important direction for future work.

Despite these considerations, EvoDiffMol presents a promising approach for molecular design that combines evolutionary optimization with 3D diffusion models. The 3D representation contributes primarily to structural validity during iterative generation; evaluating 3D-sensitive properties represents an important next step.

## Methods

This section provides a comprehensive description of the EvoDiffMol framework, including the integration of evolutionary algorithms with diffusion models, the datasets used for training and evaluation, and the performance metrics employed to assess molecular generation quality. We detail the technical implementation of the diffusion process, and evolutionary optimization strategy that enables property-driven molecular design.

### Datasets and molecular properties

Our experiments utilize the MOSES dataset [56] for model training and evaluation. MOSES consists of approximately 1.9 million structurally clean, lead-like molecules derived from the ZINC database [33], with molecular weights between 250–350 Da, fewer than 7 rotatable bonds, and XlogP values below 3.5. This dataset was specifically curated to represent drug-like chemical space with favorable pharmaceutical properties, making it suitable for evaluating generative models in molecular design applications.

In this study, we demonstrate optimization across a diverse range of molecular properties, from simple physicochemical descriptors to clinically relevant ADMET characteristics. Properties are computed using the RDKit computational chemistry toolkit [5] for basic descriptors and established ADMET prediction models for pharmacokinetic properties, ensuring consistency with benchmarks in the field. The key descriptors demonstrated include:

Lipophilicity (LogP): Quantifies the logarithm of the partition coefficient between water and octanol, measuring a molecule’s hydrophobic character. Higher LogP values indicate greater lipophilicity, which affects membrane permeability and bioavailability [4, 50].

Synthetic accessibility (SA): Evaluates the estimated difficulty of synthesizing a compound, scored from 1 (easily synthesizable) to 10 (very difficult to synthesize) [17]. This metric helps ensure generated molecules are practically accessible.

Topological polar surface area (TPSA): Calculates the sum of polar atom surface areas, predicting a molecule’s ability to cross biological membranes [18].

Quantitative estimate of drug-likeness (QED): Provides a composite drug-likeness score ranging from 0 (unfavorable properties) to 1 (optimal drug-like characteristics), integrating multiple molecular descriptors into a single metric [6].

ADMET properties: Clinically relevant pharmacokinetic descriptors predicted using the ADMET-AI machine learning package [63], which employs graph neural networks trained on experimental data. hERG (human Ether-à-go-go-Related Gene) cardiotoxicity predictions range from 0 to 1, where lower values indicate reduced risk of cardiac side effects. Caco-2 permeability predictions quantify intestinal absorption potential, where higher values indicate better oral bioavailability. These properties directly correlate with drug efficacy and safety profiles in pharmaceutical development.

### Evolutionary-diffusion integration

EvoDiffMol integrates evolutionary optimization with diffusion models through a coordinated generate-evaluate-adapt strategy that iteratively refines molecular populations toward desired property profiles. The framework operates through three key components:

Population management: The framework maintains molecular populations as collections of three-dimensional coordinate sets with associated atomic types and connectivity patterns. Population initialization employs the diffusion model to generate diverse starting configurations, ensuring broad chemical space coverage while maintaining structural validity.

Fitness evaluation: Each molecular structure undergoes comprehensive property assessment using established computational methods. Molecular properties including LogP, QED, SA, and TPSA are calculated and aggregated to produce composite fitness scores that balance multiple optimization objectives.

Evolutionary operations: The framework employs a generate-evaluate-select strategy rather than traditional genetic operators. Variation is introduced through stochastic diffusion sampling, which generates structurally diverse molecular candidates from the current model state. Selection mechanisms employ fitness-based ranking to identify superior candidates, with elite molecules forming the next generation and serving as training data for adaptive model fine-tuning.

The evolutionary framework employs a $(\mu + \lambda )$ selection strategy where newly generated molecules compete with the existing elite population for survival. Each iteration involves: (1) adaptive fine-tuning of the diffusion model on the current elite population, (2) generation of new molecular candidates using the updated model, (3) fitness evaluation of all molecules using harmonic mean aggregation of target properties, and (4) selection of the top-scoring molecules to form the next elite population.

Elite population selection follows fitness-based ranking:

1 $$\begin{aligned} P_{t+1} = \text {TopK}(P_t \cup G_t, k) \end{aligned}$$

where $P_t$ is the elite population at generation *t*, $G_t$ represents newly generated candidates, and TopK selects the *k* highest-fitness molecules from the combined population $P_t \cup G_t$.

The adaptive nature of the framework lies in its dynamic training strategy: rather than using a fixed training dataset, the diffusion model is iteratively fine-tuned on the evolving elite population. This creates a feedback loop where high-performing molecules from each generation become the training data for the next iteration, progressively biasing the model toward generating molecules with desired properties.

For hyperparameter settings please see Supporting Information.

Computational considerations: Iterative diffusion-based generation combined with evolutionary fine-tuning imposes substantial computational requirements. Each generation cycle involves model fine-tuning on elite populations followed by sampling from the adapted diffusion model, with individual molecular generation requiring 1000 denoising steps. These computational constraints necessitate careful selection of population sizes to balance optimization effectiveness with computational feasibility (see Supporting Information for experimental configurations).

### Diffusion models

EvoDiffMol employs a three-dimensional diffusion model [26–28, 60] that operates directly on molecular coordinates and atom types, enabling generation of geometrically consistent molecular structures. The implementation builds upon and extends the Molecular Diffusion Model (MDM) framework [28], adapting it for evolutionary optimization and scaffold-constrained generation. The model represents molecules as point clouds $\textbf{G} = (\textbf{R}, \textbf{A})$ where $\textbf{R} \in \mathbb {R}^{N \times 3}$ denotes atomic coordinates and $\textbf{A} \in \mathbb {R}^{N \times f}$ represents atom features including types and charges. The framework supports both unconstrained molecular generation and scaffold-constrained design through adaptive masking strategies.

Forward diffusion process: The forward process systematically corrupts molecular structures by independently perturbing coordinates and atom types through Gaussian noise addition. The forward diffusion follows a Markovian process where the probability of a complete trajectory is [27, 28]:

2 $$\begin{aligned} q(\textbf{G}_{1:T} | \textbf{G}_0) = \prod _{t=1}^{T} q(\textbf{G}_t | \textbf{G}_{t-1}) \end{aligned}$$

Each transition adds Gaussian noise with a variance schedule $\beta _1, \ldots , \beta _T$ where $\beta _t \in (0,1)$ controls the noise level at timestep *t*:

3 $$\begin{aligned} q(\textbf{G}_t | \textbf{G}_{t-1}) = \mathcal {N}(\textbf{G}_t; \sqrt{1-\beta _t}\textbf{G}_{t-1}, \beta _t\textbf{I}) \end{aligned}$$

By defining $\alpha _t = 1-\beta _t$ (signal retention coefficient) and $\bar{\alpha }_t = \prod _{s=1}^{t}\alpha _s$ (cumulative signal coefficient), the forward process admits a closed-form expression for any timestep:

4 $$\begin{aligned} q(\textbf{G}_t | \textbf{G}_0) = \mathcal {N}(\textbf{G}_t; \sqrt{\bar{\alpha }_t}\textbf{G}_0, (1-\bar{\alpha }_t)\textbf{I}) \end{aligned}$$

Here $\sqrt{\bar{\alpha }_t}$ scales the original signal while $\sqrt{1-\bar{\alpha }_t}$ scales the accumulated noise, ensuring the total variance remains constant.

This reparameterization enables direct sampling at any timestep without iterative noise addition. The forward process ensures molecular structures are progressively transformed into Gaussian noise over *T* timesteps.

Reverse denoising process: The generative process learns to reverse the corruption by predicting noise components through neural network dynamics. The complete reverse process is defined as a Markov chain:

5 $$\begin{aligned} p_\theta (\textbf{G}_{0:T}) = p(\textbf{G}_T) \prod _{t=1}^{T} p_\theta (\textbf{G}_{t-1} | \textbf{G}_t) \end{aligned}$$

where $p(\textbf{G}_T) = \mathcal {N}(\textbf{G}_T; \textbf{0}, \textbf{I})$ is the standard Gaussian prior distribution. For unconstrained generation, each reverse transition is:

6 $$\begin{aligned} p_\theta (\textbf{G}_{t-1} | \textbf{G}_t) = \mathcal {N}(\textbf{G}_{t-1}; \mu _\theta (\textbf{G}_t, t), \sigma _t^2\textbf{I}) \end{aligned}$$

where $\mu _\theta (\textbf{G}_t, t)$ is the learned mean function and $\sigma _t^2$ is the noise variance (typically fixed or learned). For scaffold-constrained generation, the reverse process is conditioned on the fixed scaffold context $\textbf{u}$:

7 $$\begin{aligned} p_\theta (\textbf{G}_{t-1} | \textbf{G}_t, \textbf{u}) = \mathcal {N}(\textbf{G}_{t-1}; \mu _\theta (\textbf{G}_t, \textbf{u}, t), \sigma _t^2\textbf{I}) \end{aligned}$$

The mean is parameterized through noise prediction following the denoising diffusion probabilistic model (DDPM) formulation:

8 $$\begin{aligned} \mu _\theta (\textbf{G}_t, \textbf{u}, t) = \frac{1}{\sqrt{\alpha _t}}\left( \textbf{G}_t - \frac{\beta _t}{\sqrt{1-\bar{\alpha }_t}}\boldsymbol{\epsilon }_\theta (\textbf{G}_t, \textbf{u}, t)\right) \end{aligned}$$

where $\boldsymbol{\epsilon }_\theta (\textbf{G}_t, \textbf{u}, t)$ is the neural network predicting the noise $\boldsymbol{\epsilon }$ added during the forward process. The coefficient $\frac{1}{\sqrt{\alpha _t}}$ rescales the noisy observation, while $\frac{\beta _t}{\sqrt{1-\bar{\alpha }_t}}$ weights the predicted noise removal. This parameterization enables stable training by predicting noise rather than the clean signal directly.

Training objective: The model is trained by optimizing the evidence lower bound (ELBO) on the data likelihood. Following the DDPM simplification, the training objective reduces to:

9 $$\begin{aligned} \mathcal {L} = \mathbb {E}_{t,\textbf{G}_0,\boldsymbol{\epsilon }} \left[ \left\| \boldsymbol{\epsilon } - \boldsymbol{\epsilon }_\theta (\textbf{G}_t, \textbf{u}, t) \right\| ^2 \right] \end{aligned}$$

where $t \sim \text {Uniform}(1, T)$ samples timesteps uniformly, $\boldsymbol{\epsilon } \sim \mathcal {N}(\textbf{0}, \textbf{I})$ is the ground-truth noise, and $\textbf{G}_t = \sqrt{\bar{\alpha }_t}\textbf{G}_0 + \sqrt{1-\bar{\alpha }_t}\boldsymbol{\epsilon }$ represents the noisy molecular state at timestep *t*. The loss function trains the network to predict the exact noise that was added during corruption.

For scaffold-constrained training, the framework applies selective masking during both forward and reverse processes. During forward diffusion, noise is only applied to variable atoms:

10 $$\begin{aligned} \textbf{G}_t = \sqrt{\bar{\alpha }_t}\textbf{G}_0 + \sqrt{1-\bar{\alpha }_t}\boldsymbol{\epsilon } \odot \textbf{m} \end{aligned}$$

where scaffold atoms ($m_i = 0$) remain unchanged. The training loss is correspondingly masked to focus only on variable atoms:

11 $$\begin{aligned} \mathcal {L}_{\text {masked}} = \mathbb {E}_{t,\textbf{G}_0,\boldsymbol{\epsilon }} \left[ \left\| (\boldsymbol{\epsilon } - \boldsymbol{\epsilon }_\theta (\textbf{G}_t, \textbf{u}, t)) \odot \textbf{m} \right\| ^2 \right] \end{aligned}$$

where $\textbf{m} \in \{0,1\}^N$ is the update mask ($m_i = 0$ for fixed scaffold atoms, $m_i = 1$ for variable atoms), $\textbf{u}$ represents the scaffold context coordinates, and $\odot$ denotes element-wise multiplication. This approach treats scaffold structures as three-dimensional context that conditions the diffusion process, similar to fragment-based design where fixed molecular fragments guide generation of connecting regions.

Sampling process: Generation begins by sampling initial noise $\textbf{G}_T \sim \mathcal {N}(\textbf{0}, \textbf{I})$ and iteratively denoising through the reverse process for *T* timesteps (typically $T = 1000$). At each timestep *t*, the neural network $\boldsymbol{\epsilon }_\theta (\textbf{G}_t, \textbf{u}, t)$ predicts the noise to be removed, and the mean of the reverse transition $\boldsymbol{\mu }_t$ is computed using the reparameterization in Equation (8). For scaffold-constrained generation, the sampling process applies selective masking at each denoising step: scaffold atoms retain their original coordinates $\textbf{u}$ while variable atoms follow the predicted denoising trajectory. This ensures structural integrity of fixed molecular fragments throughout the generation process.

### Neural network dynamics

The noise prediction network $\boldsymbol{\epsilon }_\theta (\textbf{G}_t, \textbf{u}, t)$ employs E(3)-equivariant graph neural networks (EGNN) [27] to ensure rotational and translational invariance of generated molecular structures. The network processes molecular graphs through dual edge encodings that distinguish between local chemical interactions and global intermolecular forces (van der Waals and electrostatic).

Context and timestep integration: The network incorporates both temporal and structural conditioning. Diffusion timestep information *t* is embedded through sinusoidal encoding and integrated with node features to enable timestep-dependent predictions. For scaffold-constrained generation, the fixed scaffold coordinates serve as three-dimensional context $\textbf{u}$ that conditions the diffusion process $p(\textbf{G}_{t-1} | \textbf{G}_t, \textbf{u})$. The update mask $\textbf{m}$ guides the network to predict noise only for variable atoms while preserving scaffold geometry throughout the denoising trajectory.

Dual-scale architecture: The framework employs dual EGNN encoders [28] that process molecular interactions at different scales. The global encoder operates on all atom pairs to capture long-range van der Waals interactions, while the local encoder processes only edges within a 2 Å radius to model covalent bonding patterns. The final noise prediction combines contributions from both scales through weighted summation:

12 $$\begin{aligned} \boldsymbol{\epsilon }_\theta (\textbf{G}_t, \textbf{u}, t) = w_{\text {local}} \boldsymbol{\epsilon }_{\text {local}}(\textbf{G}_t, \textbf{u}, t) + w_{\text {global}} \boldsymbol{\epsilon }_{\text {global}}(\textbf{G}_t, \textbf{u}, t) \end{aligned}$$

where $\boldsymbol{\epsilon }_\theta (\textbf{G}_t, \textbf{u}, t)$ represents the combined noise prediction for both coordinates and node features, and $w_{\text {local}}$, $w_{\text {global}}$ are weighting factors that balance the contributions from different interaction scales during sampling.

Equivariant updates: Each encoder updates both node features and coordinates while preserving E(3)-equivariance. The coordinate updates depend linearly on relative positions:

13 $$\begin{aligned} \Delta \textbf{r}_i = \sum _{j \in \mathcal {N}(i)} \phi _r(\textbf{h}_i, \textbf{h}_j, d_{ij}^2) \cdot \frac{\textbf{r}_i - \textbf{r}_j}{d_{ij}} \end{aligned}$$

where $\phi _r$ represents learnable functions parameterized by multi-layer perceptrons, $\textbf{h}_i$ denotes node embeddings, $d_{ij}$ is the Euclidean distance, and $\mathcal {N}(i)$ defines the neighborhood set. The position displacements depend linearly on coordinate differences, ensuring geometric equivariance under rotations and translations.

### Performance evaluation

EvoDiffMol performance assessment employs three fundamental metrics that capture different aspects of molecular generation quality:

Validity: Measures the fraction of generated molecular structures that conform to standard chemical rules and constraints. Each generated molecule is evaluated using RDKit to determine whether it satisfies proper atomic valency requirements (e.g., carbon forms four bonds) and represents a chemically feasible structure. Invalid structures are those with impossible bonding patterns, such as pentavalent carbon or incorrect charge states.

Uniqueness: Quantifies structural diversity within generated molecular sets by calculating the percentage of distinct valid molecules present in the output population.

Novelty: Evaluates the originality of generated molecules by determining the fraction of unique valid structures that are absent from the original training dataset. Generated molecules are converted to canonical SMILES representations and compared against the complete training set to identify previously unseen chemical structures.

Fitness score: For multi-property optimization, composite fitness scores aggregate performance across multiple objectives. For *n* normalized property values $p_1, p_2, \ldots , p_n$, the harmonic mean provides robust aggregation:

14 $$\begin{aligned} F = \frac{n}{\sum _{i=1}^{n} \frac{1}{p_i}} \end{aligned}$$

This formulation ensures that evolutionary optimization targets well-balanced molecular candidates that satisfy multiple design criteria simultaneously, rather than optimizing individual properties at the expense of others. Complete hyperparameter settings and implementation details are provided in the Supporting Information.

## Supplementary Information

Supplementary material 1.

## Data Availability

The MOSES dataset used in this study is publicly available at https://github.com/molecularsets/moses.

## Code availability

Source code for the EvoDiffMol framework is available at https://github.com/XiaoboLinlin/EvoDiffMol under the MIT license. Pre-trained model checkpoints are available on Hugging Face at https://huggingface.co/scofieldlinlin/EvoDiffMol.
