# Supplementary material for: EvoDiffMol: evolutionary diffusion framework for 3D molecular design with optimized properties
Source: J Cheminform. 2026 Jul 15;18:98. doi: 10.1186/s13321-026-01237-y (PMC13374194; doi:10.1186/s13321-026-01237-y)
Supplement: Supplementary file 1 — Supplementary material 1. [file 13321_2026_1237_MOESM1_ESM.pdf]

EvoDiffMol: Evolutionary Diffusion Framework for 3D Molecular Design with Optimized Properties

Xiaobo Lin[1*], Logan T. Kearney[1*], Zhaoqian Su[2], Yunchao Liu[3], Amit K. Naskar[1*], Debsindhu Bhowmik[4*]

[1]Carbon and Composites Group, Chemical Sciences Division, Oak Ridge National Laboratory, Oak Ridge, 37831, Tennessee, United States.
[2]Research, Takeda Pharmaceutical Company Ltd., Cambridge, 02139, Massachusetts, United States.
[3]Broad Institute of MIT and Harvard, Cambridge, 02142, Massachusetts, United States.
[4]Computational Sciences and Engineering Division, Oak Ridge National Laboratory, Oak Ridge, 37831, Tennessee, United States.

*Corresponding author(s). E-mail(s): linx1@ornl.gov; kearneylt@ornl.gov; naskarak@ornl.gov; bhowmikd@ornl.gov;

Contents

Section S1 Hyperparameters and Implementation Details
Section S2 Technical Implementation Details
Section S3 Single Property Optimization Results
Section S4 Scaffold-Constrained ADMET Optimization Results
Section S5 Additional Analyses for Revised Manuscript

1 Hyperparameters and Implementation Details

This section provides the complete hyperparameter settings and implementation details for reproducibility of the EvoDiffMol framework.

1.1 Experimental Configurations

Different experiments in this study employed varying population sizes optimized for their specific objectives. Table 1 summarizes the experimental configurations used throughout this work.

Table 1 Experimental configurations for different optimization tasks

Experiment	Population Size	Generations	Application
QED Optimization (Table 1)	2500	10	Table 1 comparison with state-of-the-art methods
ADMET Optimization (Figure 6)	2500	10	Multi-property optimization with ADMET properties
Multi-Property Optimization (Figures 4, 5)	250	15	All property optimization experiments (single, dual, triple properties)
Scaffold-Constrained Generation (Figure 7)	250	15	Property optimization with fixed substructures

QED Optimization (Table 1): The baseline (Generation 0) represents 2,500 molecules sampled from the pre-trained diffusion model without evolutionary optimization, achieving 99% validity, 97% uniqueness, and 96% novelty. The optimized population (Generation 10) achieves 100% validity and uniqueness due to quality control mechanisms in the evolutionary loop that filter invalid molecules and duplicates, ensuring all optimized candidates are chemically valid and structurally unique. Novelty remains at 95%, confirming the framework generates new molecules rather than memorizing training data.

Multi-Property and Scaffold-Constrained Experiments: The smaller population size (250 molecules) balances computational cost with optimization effectiveness, enabling efficient exploration across multiple experimental cases including various property combinations, different target values, and scaffold constraints. This configuration is particularly advantageous when conducting extensive experimental campaigns with numerous optimization scenarios.

2 Technical Implementation Details

This section describes the technical architecture and algorithmic details of the EvoDiffMol framework, complementing the hyperparameter specifications in Section 1.

Table 2 EvoDiffMol framework hyperparameters

Parameter	Symbol	Value
**Evolutionary Algorithm Parameters**		
Elite population size	μ	250–2500*
Batch size	–	128
Scale factor (unconstrained)	–	2.0
Scale factor (scaffold)	–	2.5
Adaptive training	–	True
Use harmonic mean	–	True
**Diffusion Model Parameters**		
Hidden dimension	–	256
Number of convolutions	–	6
Number of diffusion timesteps	T	1000
Beta schedule	–	sigmoid
Beta start	β_{start}	1×10^{-7}
Beta end	β_{end}	2×10^{-3}
Learning rate	α	1×10^{-4}
**Sampling Parameters**		
Sampling type	–	generalized
Step learning rate	–	1×10^{-6}
Global position weight	$w_{\text{global,pos}}$	1.0
Global node weight	$w_{\text{global,node}}$	4.0
Local position weight	$w_{\text{local,pos}}$	1.0
Local node weight	$w_{\text{local,node}}$	5.0

*Population size varies by experiment: 2500 for QED comparison (Table 1), 250 for multi-property optimization experiments (see Experimental Configurations above).

The EvoDiffMol framework utilizes EGNN (Equivariant Graph Neural Networks) as the backbone architecture for the diffusion model, ensuring rotational and translational equivariance essential for 3D molecular generation. The model employs a sigmoid noise schedule for the diffusion process, which provides smooth transitions between noise levels and improved sampling quality compared to linear schedules.

For scaffold-constrained generation, the framework implements masked diffusion where scaffold atoms are held fixed throughout the denoising process. The update mask mechanism ensures that only non-scaffold atoms are updated during reverse diffusion, maintaining the structural integrity of the desired substructure while allowing optimization of surrounding molecular regions.

The evolutionary algorithm employs a $(\mu + \lambda)$ selection strategy, where the elite population of size μ competes with λ newly generated molecules for survival in each generation. The scale factor λ/μ varies by experiment (2.0 for unconstrained, 2.5 for scaffold-constrained), balancing exploration of new chemical space with exploitation of high-fitness molecular candidates.

3 Single Property Optimization Results

This section presents detailed single property optimization results for both unconstrained and scaffold-constrained molecular generation, providing comprehensive

analysis beyond the multi-property results shown in the main text. While the main text focuses on multi-property optimization capabilities, these results demonstrate EvoDiffMol's precise control over individual molecular descriptors.

3.1 Unconstrained Molecular Generation

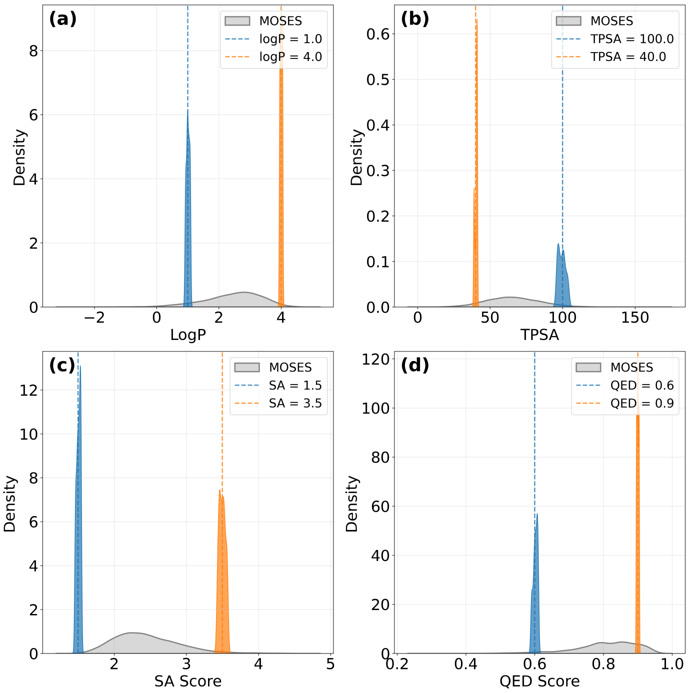

Fig. 1 Single property optimization for unconstrained molecular generation. Kernel density distributions show EvoDiffMol-generated molecules (colored) concentrated around specified target values (dashed lines) compared to the baseline MOSES dataset (gray). (a) LogP, (b) TPSA, (c) SA, (d) QED.

Single-property optimization results (Figure 1) demonstrate EvoDiffMol's precise control over individual molecular descriptors. Each panel overlays the kernel density estimation of MOSES test molecules (gray) with EvoDiffMol outputs optimized for specific targets (colored). Dashed vertical lines mark the specified targets and empirical means of generated populations. For LogP targets (1.0 and 4.0), TPSA targets (40 and 100), SA targets (1.5 and 3.5), and QED targets (0.6 and 0.9), the optimized distributions are narrow and precisely centered at the targets, while MOSES remains broadly distributed. Targets near distribution boundaries where prior density is low prove more challenging than targets in high-density regions. High TPSA ($\approx$100) or low QED ($\approx$0.6) represent boundary targets with sparse prior mass, yet EvoDiffMol successfully concentrates narrow peaks at these difficult locations. Conversely, targets in denser regions of the prior distribution facilitate easier optimization.

3.2 Scaffold-Constrained Molecular Generation

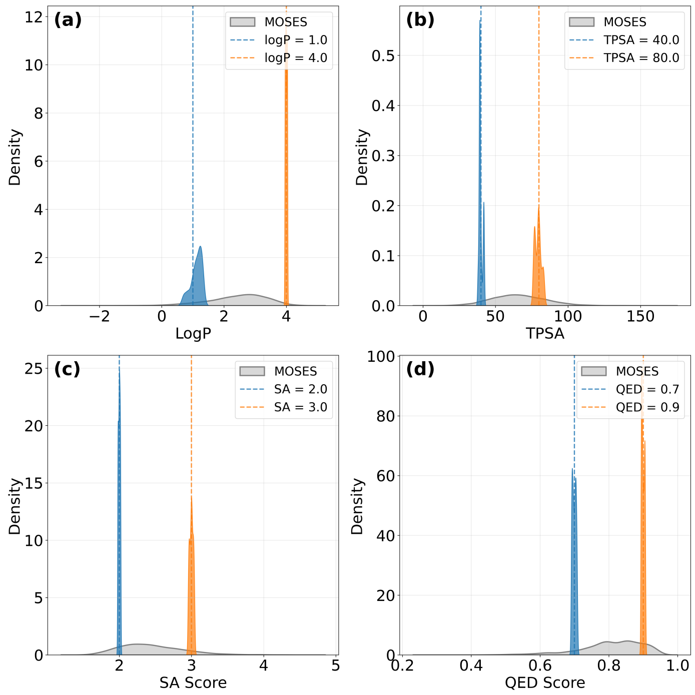

Fig. 2 Single property optimization for scaffold-constrained molecular generation with quinoline scaffold. Kernel density distributions show EvoDiffMol-generated molecules (colored) concentrated around specified target values (dashed lines) compared to the baseline MOSES dataset (gray). (a) LogP, (b) TPSA, (c) SA, (d) QED.

Single-property optimization under scaffold constraints (Figure 2) demonstrates EvoDiffMol's ability to shift generated distributions from the MOSES reference (gray) to narrow, unimodal peaks centered at user-specified targets (colored). Across LogP, TPSA, SA, and QED properties, the target-conditioned samples concentrate tightly around desired values. The separation between optimized peaks and the broad MOSES background demonstrates precise, controllable property steering under structural constraints.

4 Scaffold-Constrained ADMET Optimization Results

This section extends the scaffold-constrained optimization demonstration from the main text (which focused on quinoline with LogP, TPSA, and SA properties) to clinically relevant ADMET endpoints. We present multi-property ADMET optimization results for three pharmaceutically relevant scaffolds: piperazine, pyridine, and thiophene. These scaffolds are commonly found in drug molecules and serve as structural

constraints during molecular generation. The optimization simultaneously targets three key ADMET properties: QED, SA, and hERG toxicity.

4.1 Experimental Setup

For each scaffold, EvoDiffMol was configured to optimize a population of 500 molecules over 15 evolutionary generations. The optimization targets were set to maximize QED (target = 1.0), minimize SA score (target = 1.0, normalized), and minimize hERG toxicity (target = 0.0). The baseline comparison uses 2000 randomly sampled molecules from the MOSES test dataset, with ADMET properties calculated using the ADMET-AI model.

4.2 Results Summary

Table 3 Scaffold-constrained ADMET optimization results compared to scaffold-matched MOSES baseline

Scaffold	Property	Scaffold-Matched Baseline	EvoDiffMol	Improvement
Piperazine	QED	0.830 ± 0.069	0.831 ± 0.044	+0.1%
	SA	2.369 ± 0.401	2.551 ± 0.396	−7.7%
	hERG toxicity	0.539 ± 0.258	0.107 ± 0.054	+80.2%
Pyridine	QED	0.790 ± 0.099	0.894 ± 0.031	+13.2%
	SA	2.454 ± 0.418	2.115 ± 0.285	+13.8%
	hERG toxicity	0.418 ± 0.238	0.061 ± 0.034	+85.5%
Thiophene	QED	0.801 ± 0.096	0.877 ± 0.045	+9.5%
	SA	2.552 ± 0.418	2.636 ± 0.404	−3.3%
	hERG toxicity	0.365 ± 0.208	0.100 ± 0.054	+72.6%

Scaffold-matched baselines: MOSES test set molecules containing the target scaffold (Piperazine: 7,964; Pyridine: 34,464; Thiophene: 12,327 molecules). hERG improvements are computed against scaffold-matched baselines to isolate the effect of evolutionary optimization from intrinsic scaffold properties. ADMET properties predicted using ADMET-AI; results should be interpreted as predictor-specific trends.

Table 3 summarizes the optimization results for all three scaffolds. The most significant improvements are observed for hERG cardiotoxicity, with reductions of 71.79%, 83.94%, and 73.54% for piperazine, pyridine, and thiophene respectively, compared to the MOSES baseline. This demonstrates EvoDiffMol's ability to generate molecules with substantially lower cardiotoxicity risk while maintaining structural constraints.

For QED optimization, pyridine shows the strongest improvement (+11.36%), followed by thiophene (+9.27%) and piperazine (+3.49%). SA improvements vary by scaffold: pyridine achieves a 14.01% reduction (lower SA score is better), while piperazine and thiophene show slight increases in SA score, indicating these scaffolds may present greater synthetic challenges when constrained to specific property targets.

Figures 3, 4, and 5 show the kernel density distributions for each scaffold, comparing optimized EvoDiffMol populations against the MOSES baseline. Across all three scaffolds, the hERG toxicity distributions show the most dramatic shifts, with optimized populations concentrated at much lower values (near 0.0) compared to the broad

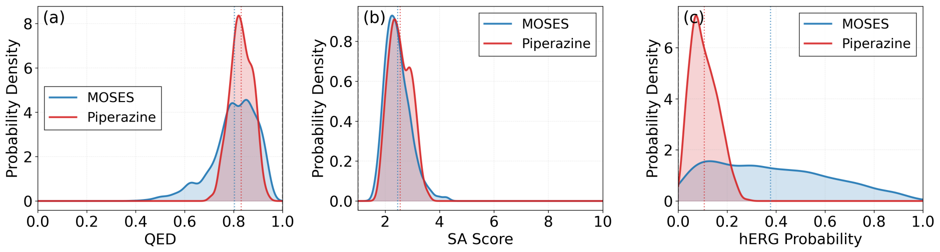

Fig. 3 ADMET property distributions for piperazine-constrained optimization. Kernel density distributions compare EvoDiffMol-generated molecules (red) against MOSES baseline (blue) for (a) QED, (b) SA, and (c) hERG toxicity. Dashed lines indicate target values and mean values for each distribution.

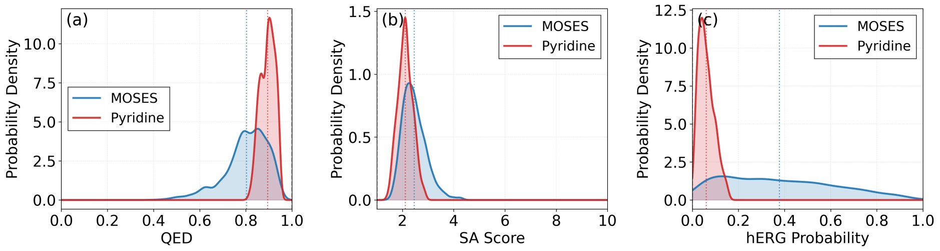

Fig. 4 ADMET property distributions for pyridine-constrained optimization. Kernel density distributions compare EvoDiffMol-generated molecules (red) against MOSES baseline (blue) for (a) QED, (b) SA, and (c) hERG toxicity. Dashed lines indicate target values and mean values for each distribution.

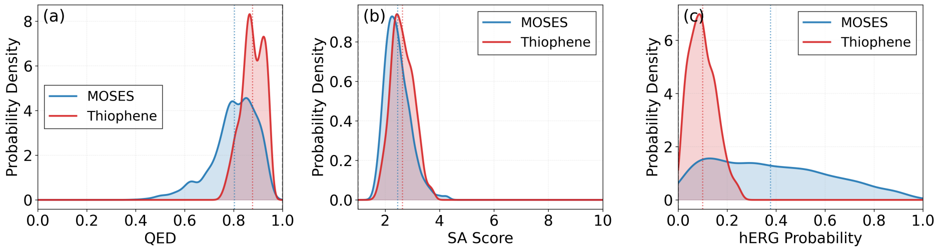

Fig. 5 ADMET property distributions for thiophene-constrained optimization. Kernel density distributions compare EvoDiffMol-generated molecules (red) against MOSES baseline (blue) for (a) QED, (b) SA, and (c) hERG toxicity. Dashed lines indicate target values and mean values for each distribution.

MOSES distribution centered around 0.38. This substantial reduction in cardiotoxicity risk is critical for drug safety.

The QED distributions demonstrate successful optimization toward higher values, with pyridine showing the tightest distribution and highest mean. SA distributions show varying degrees of optimization success, with pyridine achieving the best results (lower scores indicating easier synthesis), while piperazine and thiophene show distributions shifted toward slightly higher SA scores, reflecting the trade-offs inherent in multi-property optimization under structural constraints.

The scaffold-constrained ADMET optimization results demonstrate several important capabilities of the EvoDiffMol framework. EvoDiffMol successfully optimizes multiple ADMET properties simultaneously while maintaining fixed scaffold structures, demonstrating the framework's ability to balance competing objectives. The most significant achievement is the substantial reduction in hERG cardiotoxicity risk (71–84% improvement), which is critical for drug safety and represents a major challenge in drug design. Different scaffolds show varying optimization success rates, with pyridine achieving the best overall performance across all three properties, highlighting the importance of scaffold selection in drug design workflows.

The results reveal inherent trade-offs in multi-property optimization, where improvements in some properties (e.g., hERG) may come at the cost of others (e.g., SA for piperazine and thiophene), demonstrating the need for careful objective function design. These results validate EvoDiffMol's capability to generate drug-like molecules with optimized ADMET profiles under structural constraints, providing a powerful tool for scaffold-based drug design.

5 Additional Analyses for Revised Manuscript

This section presents additional analyses conducted during revision to address reviewer comments regarding metric transparency, quantitative evaluation, and reproducibility.

5.1 ADMET Endpoint Analysis

Table 4 ADMET endpoint analysis: optimized and non-optimized properties

Property	MOSES	EvoDiffMol	Direction
Optimized properties (targeted during evolution):			
hERG (↓)	0.370 ± 0.237	0.126 ± 0.081	Improved
Caco-2 (↑)	-4.684 ± 0.340	-3.858 ± 0.124	Improved
Non-optimized properties (evaluated post-hoc):			
CYP2D6 inhibition (↓)	0.128 ± 0.182	0.027 ± 0.041	Improved
DILI risk (↓)	0.658 ± 0.272	0.509 ± 0.285	Improved
BBB permeation (↑)	0.903 ± 0.105	0.983 ± 0.026	Improved
HIA (↑)	0.999 ± 0.006	1.000 ± 0.000	Maintained

All predictions from ADMET-AI. Non-optimized endpoints were not part of the fitness function and were evaluated post-hoc to assess whether the optimization produces generally favorable drug-like molecules. MOSES baseline: 2,000 randomly sampled test molecules. EvoDiffMol: optimized population from QED+SA+hERG+Caco-2 experiment (2,500 molecules). ADMET predictions are predictor-specific; different predictors may yield different results.

5.2 Cross-Generation Novelty and Uniqueness

Table 5 Cross-generation novelty and uniqueness metrics across evolutionary generations

Generation	MOSES-only Novelty	Cross-gen Novelty	Cross-gen Uniqueness
0 (baseline)	96.1%	96.1%	100%
1	93.3%	60.0%	65.0%
2	92.6%	38.5%	41.3%
3	93.3%	24.2%	25.6%
4	94.4%	21.3%	22.2%
5	94.4%	18.2%	19.3%
6	95.0%	15.4%	16.2%
7	95.0%	12.3%	12.8%
8	95.3%	11.0%	11.4%
9	95.3%	10.4%	10.6%
10 (final)	95.2%	9.4%	9.7%

MOSES-only novelty: fraction of molecules not present in the MOSES training set. Cross-generation novelty: fraction of molecules not present in MOSES training set *or* any previous generation's elite population. Cross-generation uniqueness: fraction of molecules in generation t not seen in any generation 0 through $t-1$. The decrease in cross-generation novelty reflects the intended behavior of adaptive fine-tuning: the model progressively generates molecules similar to high-performing elites. MOSES-only novelty remains stable at 93–95%, confirming no memorization of training data. Overall: 8,346 unique molecules out of 27,493 total across all generations (30.4% cross-generation uniqueness). QED optimization, population size 2,500, seed 2025.

5.3 Multi-Seed Reproducibility

Table 6 Multi-seed reproducibility analysis for QED optimization

Seed	QED	Validity	Uniqueness	Novelty	Scaff. Div.	Tanimoto
2025	0.936 ± 0.007	100%	100%	95.1%	74.7%	0.172
42	0.935 ± 0.007	100%	100%	95.7%	73.3%	0.175
123	0.936 ± 0.006	100%	100%	95.6%	71.4%	0.170
7777	0.936 ± 0.006	100%	100%	95.6%	73.0%	0.174
**Mean**	**0.936**	**100%**	**100%**	**95.5%**	**73.1%**	**0.173**
**Std**	**0.001**	**0%**	**0%**	**0.2%**	**1.2%**	**0.002**

Post-selection elite population metrics. All runs use identical configuration: population size 2,500, 10 generations, QED target 1.0, scale factor 2.0. QED values report within-population mean ± standard deviation. Scaffold diversity: percentage of unique Bemis-Murcko scaffolds. Tanimoto: mean pairwise similarity (10,000 random pairs). Between-run QED standard deviation of 0.001 confirms robust convergence.

Table 7 Raw generation metrics before selection (seed 42)

Generation	Attempted	Raw Validity	Raw Uniqueness
0 (initial)	5,000	99.1% (4,956)	99.3% (4,923)
1	5,000	99.4% (4,969)	96.4% (4,788)
3	5,000	99.5% (4,976)	92.0% (4,577)
5	5,000	99.7% (4,984)	93.8% (4,676)
7	5,000	99.6% (4,981)	91.3% (4,549)
10 (final)	5,000	99.8% (4,989)	92.0% (4,588)

Raw metrics represent all molecules generated by the diffusion model *before* fitness-based elite selection. Validity: fraction of generated molecules that are chemically valid. Uniqueness: fraction of valid molecules with unique SMILES (within the same generation batch). Raw validity remains ∼99% throughout optimization. Raw uniqueness decreases from 99.3% to ∼92% as the fine-tuned model converges, but remains above 90%. Similar patterns observed across all seeds.

5.4 Quantitative Multi-Property Optimization Metrics

Table 8 Quantitative multi-property optimization metrics

Type	Targets	MAD	Hit Rate	Joint Success
Single property (9 experiments):				
Single	LogP=1.0	0.046	100%	—
Single	LogP=4.0	0.031	100%	—
Single	QED=0.6	0.006	100%	—
Single	QED=0.9	0.002	100%	—
Single	QED=1.0*	0.061	0%*	—
Single	SA=1.5	0.024	100%	—
Single	SA=3.5	0.036	100%	—
Single	TPSA=40	0.850	100%	—
Single	TPSA=100	2.185	100%	—
Double property (10 experiments):				
Double	LogP=1, QED=0.9	0.17, 0.02	100%, 96%	96.0%
Double	LogP=1, SA=2	0.14, 0.18	100%, 100%	99.6%
Double	LogP=1, TPSA=40	0.23, 5.93	94%, 80%	73.6%
Double	LogP=3, SA=3	0.14, 0.14	100%, 100%	100%
Double	LogP=3, TPSA=80	0.30, 4.91	80%, 96%	76.4%
Double	LogP=4, QED=0.6	0.25, 0.02	92%, 99%	90.8%
Double	TPSA=40, QED=0.9	1.55, 0.01	100%, 100%	100%
Double	TPSA=40, SA=1.5	1.58, 0.11	100%, 100%	100%
Double	TPSA=100, SA=3.5	4.69, 0.28	92%, 86%	78.0%
Double	TPSA=100, QED=0.6	8.00, 0.04	64%, 65%	31.2%
Triple property (2 experiments):				
Triple	LogP=2.5, TPSA=40, SA=2	0.16, 3.3, 0.14	100%, 99%, 98%	97.6%
Triple	LogP=1, TPSA=80, SA=3	0.21, 3.9, 0.29	97%, 96%, 84%	77.2%

MAD: mean absolute deviation between target and population mean. Hit rate: fraction of molecules within tolerance (±0.5 for LogP, ±10 for TPSA, ±0.5 for SA, ±0.05 for QED). Joint success: fraction simultaneously satisfying all tolerances. *QED=1.0 is the theoretical maximum; no molecule reaches the ±0.05 tolerance window. Population size 250, 15 generations.

5.5 Scaffold Integrity Retention Rates

Table 9 Scaffold integrity retention rates for scaffold-constrained generation

Scaffold	Attempted /gen	Valid + Scaffold Intact Early (Gen 1–5)	Late (Gen 11–15)	Post-selection integrity
Pyridine	1,250	853 (68%)	908 (73%)	500/500 (100%)
Thiophene	1,250	293 (23%)	428 (34%)	500/500 (100%)
Piperazine	1,250	1,176 (94%)	1,176 (94%)	500/500 (100%)

"Valid + Scaffold Intact" counts molecules that are chemically valid and contain the target scaffold with correct connectivity (verified by SMARTS substructure matching). Early/Late columns show mean counts for generations 1–5 and 11–15, respectively; the improvement for thiophene (23% → 34%) demonstrates that adaptive fine-tuning progressively learns scaffold-compatible geometries. Attempted/gen = elite_size × scale_factor = 500 × 2.5. Post-selection integrity verified by SMARTS matching with aromaticity checks. Scaffolds studied lack chiral centers; stereochemistry preservation not applicable.
